# Glucose metabolism is upregulated in the mononuclear cell proteome during sepsis and supports endotoxin-tolerant cell function

**DOI:** 10.3389/fimmu.2022.1051514

**Published:** 2022-11-18

**Authors:** Bianca Lima Ferreira, Mônica Bragança Sousa, Giuseppe Gianini Figueirêdo Leite, Milena Karina Colo Brunialti, Erika Sayuri Nishiduka, Alexandre Keiji Tashima, Tom van der Poll, Reinaldo Salomão

**Affiliations:** ^1^ Division of Infectious Diseases, Escola Paulista de Medicina, Universidade Federal de São Paulo, São Paulo, Brazil; ^2^ Department of Biochemistry, Escola Paulista de Medicina, Universidade Federal de São Paulo, São Paulo, Brazil; ^3^ Center of Experimental and Molecular Medicine, Amsterdam University Medical Centers (UMC), University of Amsterdam, Amsterdam, Netherlands; ^4^ Division of Infectious Diseases, Amsterdam Amsterdam University Medical Centers (UMC), University of Amsterdam, Amsterdam, Netherlands

**Keywords:** sepsis, immunometabolism, PBMCs, endotoxin-tolerance, LPS, glycolysis, pentose phosphate pathway

## Abstract

Metabolic adaptations shape immune cell function. In the acute response, a metabolic switch towards glycolysis is necessary for mounting a proinflammatory response. During the clinical course of sepsis, both suppression and activation of immune responses take place simultaneously. Leukocytes from septic patients present inhibition of cytokine production while other functions such as phagocytosis and production of reactive oxygen species (ROS) are preserved, similarly to the *in vitro* endotoxin tolerance model, where a first stimulation with lipopolysaccharide (LPS) affects the response to a second stimulus. Here, we sought to investigate how cellular metabolism is related to the modulation of immune responses in sepsis and endotoxin tolerance. Proteomic analysis in peripheral blood mononuclear cells (PBMCs) from septic patients obtained at intensive care unit admission showed an upregulation of proteins related to glycolysis, the pentose phosphate pathway (PPP), production of ROS and nitric oxide, and downregulation of proteins in the tricarboxylic acid cycle and oxidative phosphorylation compared to healthy volunteers. Using the endotoxin-tolerance model in PBMCs from healthy subjects, we observed increased lactate production in control cells upon LPS stimulation, while endotoxin-tolerant cells presented inhibited tumor necrosis factor-α and lactate production along with preserved phagocytic capacity. Inhibition of glycolysis and PPP led to impairment of phagocytosis and cytokine production both in control and in endotoxin-tolerant cells. These data indicate that glucose metabolism supports leukocyte functions even in a condition of endotoxin tolerance.

## 1 Introduction

The regulation of inflammatory responses is strongly related to metabolic adaptations in immune cells (1). During acute inflammation, the metabolic switch from oxidative phosphorylation (OXPHOS) to aerobic glycolysis (Warburg effect) allows activated leukocytes to obtain a rapid source of ATP through pyruvate conversion to lactate, while providing metabolic intermediates to support cell proliferation, cytokine production, and antimicrobial responses (1). However, in later phases, there is a switch to fatty acid oxidation and a transition to a catabolic state, related to reduced inflammation (2).

Sepsis is defined as a life-threatening organ dysfunction caused by a dysregulated host response to infection (3), which is related to marked cell metabolism changes (4). Sepsis is a major global health problem: in 2017 there was an estimated total of almost 50 million incident cases of sepsis and 11 million deaths worldwide (5). Both suppression and exacerbation of immune responses are present concomitantly in the clinical continuum of sepsis (6, 7). Return to homeostasis is associated with a quick recovery, while the presence of a persistent inflammation, immunosuppression, and catabolism syndrome (PICS) is related to a prolonged ICU stay and a worse prognosis (6, 8). The concurrent inhibition and activation of immune response can be observed in sepsis patients’ monocytes, which present reduced HLA-DR expression and a diminished ability to produce inflammatory cytokines upon *ex vivo* stimulation, while maintaining the capacity to phagocytose and to produce reactive oxygen species (ROS) and nitric oxide (NO) (7, 9, 10). This phenotype is similar to the immunomodulation observed in *in vitro* endotoxin-tolerance, where cells pre-stimulated with lipopolysaccharide (LPS) present an impaired inflammatory cytokine production upon challenge with LPS or other agonists, although preserving ROS production and phagocytosis (7, 11, 12).

We have previously described a distinct regulation of gene transcription related to the metabolic regulator hypoxia-inducible factor (HIF)-1α and glycolysis in septic patients depending on the clinical course (13). It has been described that endotoxin-tolerant leukocytes from patients with sepsis present a broad inhibition in cellular metabolism (14), and monocytes from healthy volunteers submitted to *in vivo* endotoxemia displayed a loss of metabolic plasticity after *ex vivo* stimulation with LPS (15).

Additionally, we have recently found altered carbon metabolism as an important module common to the transcriptome and proteome of septic patients’ leukocytes. We also observed a network comprising downregulated genes/proteins related to mitochondrial translation (16). Here, to further investigate how cellular metabolism is related to immunomodulation in sepsis, we performed a metabolic pathway-targeted analysis using this previously published proteomic dataset (16) with peripheral blood mononuclear cell (PBMC) samples obtained from patients at intensive care unit (ICU) admission and included samples obtained after seven days of enrollment from the same cohort. To support the obtained proteomic results, we assessed metabolic and immune cellular changes in the endotoxin-tolerance model and used metabolic pathway inhibitors to evaluate how metabolism affects the modulated immune response in endotoxin-tolerant leukocytes.

## 2 Materials and methods

### 2.1 Proteomic analysis

#### 2.1.1 Collection of proteomic data

Proteomic data of PMBCs from septic patients and healthy controls were obtained from ProteomeXchange Consortium/MassIVE repository MSV000087733 (16). This dataset includes samples from 24 patients with sepsis secondary to community-acquired infections obtained within 48 hours of ICU admission (D0). As previously reported, 45.8% were male and the mean age was 70.2 (SD± 14.4) years. Ten (41.7%) patients presented with septic shock and 5 (20.8%) died during their hospital stay. SOFA at admission was 5.52 (SD± 3.20), respiratory and renal dysfunctions were presented in 33.3% and 29.2% of the patients, respectively (16). The dataset also included 9 healthy volunteers as the control group. Additionally, PBMCs stored in the biobank BR047 (CEP/UNIFESP-HSP) obtained after 7 days of enrollment (D7) from 17 patients in the same cohort of the published dataset were included in this study. Written informed consent was obtained from all participants before blood sampling or, when this was not possible, from relatives. This study was approved by the ethics committees of participating hospitals and the use of stored samples was approved by the institutional ethics committee (CEP/UNIFESP 1171/2017).

#### 2.1.2 Protein sample preparation, mass spectrometry acquisition, and label-free quantification

Sample preparation, LC-MS/MS analysis in the chromatographic system nanoAcquity ultra-performance liquid chromatography (UPLC) (Waters, Milford, MA) coupled to the Synapt G2 HDMS Mass Spectrometer (Waters) and label-free quantification using Progenesis QI (NonLinear Dynamics, Newcastle upon Tyne, UK) of PBMC samples from patients at D7 were carried out simultaneously with samples from the dataset MSV000087733, as previously described (16).

#### 2.1.3 Bioinformatic analysis of proteomics data

Progenesis output tables were imported and analyzed in Perseus software (v. 1.6.15.0) (17). The normalized data were log2 transformed and intensity values were filtered to have at least 60% quantified values in one or more of the groups (Sepsis D0, Sepsis D7, or Healthy). Proteins were considered differentially abundant in patients when Student’s t-test p-value < 0.05 and Log_2_FC < −0.37 or > 0.37.

Gene Ontology Biological Process (GOBP) analysis in D0 and D7 data was performed in the Molecular Signatures Database (MsigDB v. 7.4) with a false discovery rate (FDR) < 0.05 (18). Altered metabolic pathways were identified for both groups with Ingenuity Pathway Analysis (IPA, Qiagen, Hilden, Germany), metabolism-related pathways were filtered from canonical pathways with a BH corrected p-value < 0.05 and a z-score as a predictor of inhibition or activation. GOBP and IPA data were imported to R software (v. 4.0.2) for comparison between D0 and D7 groups and graphs were generated with ggplot2 (v. 3.3.3). Schematic representations of metabolic pathways were generated with Escher (https://escher.github.io/) (19).

### 2.2 *In vitro* endotoxin-tolerance assays

#### 2.2.1 Isolation of PBMCs

Recruitment of healthy volunteers for blood sampling was approved by the institutional ethics committee (CEP/UNIFESP 1172/2020) and all donors gave informed consent before inclusion in the study. The average age was 32.8 years (SD ±11.2) and 60% were female. Blood was diluted in PBS (1:1, v:v) and PBMCs were isolated by the Ficoll gradient method (Ficoll-Paque PLUS; GE Healthcare Bio-Sciences, Uppsala, Sweden). Isolated cells were cultured in RPMI 1640 medium (Gibco, Thermo Fisher Scientific, Waltham, MA) supplemented with 2 mM L-glutamine (Gibco), 1 mM sodium pyruvate (Gibco), 10 mM HEPES (Gibco), 55 µM 2-Mercaptoethanol (Gibco) and 10% Human AB serum (H6914, Sigma-Aldrich, Saint Louis, MO) in polypropylene tubes to avoid cell adhesion.

#### 2.2.2 Induction of endotoxin-tolerance

To induce endotoxin-tolerance in PBMCs 2.5x10^6^ cells/mL were incubated in the presence or absence (control) of lipopolysaccharide (LPS) from *Salmonella abortus equi* (kindly provided by C. Galanos, Max-Planck Institute of Immunobiology, Germany) at 10 ng/mL or 100 ng/mL for 48 hours (h) at 37 °C and 5% CO_2_. Tolerant and naïve control cells were then washed twice in PBS and challenged with 100 ng/mL LPS for 24h at 37 °C and 5% CO_2_; a group cultured only in supplemented RPMI (both incubation times) was included as a negative control.

#### 2.2.3 Detection of intracellular tumor necrosis factor-α in monocytes by flow cytometry

For intracellular cytokine measurement, 10 µg/mL brefeldin A (Thermo Fisher Scientific, Waltham, MA) was added to the cell suspension after 30 minutes (min) of incubation with the challenge dose of LPS. After 24 hours, cells were washed and stained with anti-CD14-BV711, clone MφP9 (BD Biosciences, Franklin Lakes, NJ) for identification of monocytes. Intracellular staining with anti-TNF-α-PE-Cy7, clone Mab11 (BD Biosciences) was performed after fixation and permeabilization with 2× lysing solution (BD Biosciences) containing 0.05% Tween 20. The cells were then washed and suspended in a buffer solution (PBS, 0.1% BSA, 2 mM EDTA) before flow cytometric analysis; the gating strategy is described in **Supplementary File 1**. Flow cytometry was performed in an LSR FORTESSA (BD Biosciences), and data were analyzed using the FlowJo software (FlowJo v10, BD Biosciences).

#### 2.2.4 Quantification of ATP and lactate

After 24h of LPS challenge, cells were spun down, supernatants were stored for lactate quantification and cell pellets were lysed for ATP measurement with ATP Assay Kit Colorimetric/Fluorimetric (Abcam, Cambridge, United Kingdom). Briefly, after lysis samples were centrifuged at 16000 g for 2 min at 4°C, and an aliquot of cell lysate was stored for total protein quantification using the Bradford method. Samples were then deproteinized by centrifugation in 10kD spin columns (Abcam) at 10000 g for 40 min at 4°C, snap-frozen in liquid nitrogen, and stored at -80°C. ATP measurement was carried out using the fluorometric method following the manufacturer’s instructions, fluorescence was measured in a Synergy H1 plate reader (Biotek, Winooski, VT) at Ex/Em of 535/587nm and results were calculated as pmol ATP/µg protein. Lactate was measured in supernatants using a colorimetric commercial kit, following the manufacturer’s instructions (Labtest, Lagoa Santa-MG, Brazil).

#### 2.2.5 Assessment of cytokine production and phagocytosis capacity after metabolic modulation

Endotoxin tolerance was induced as previously described: 2.5x10^6^ PBMCs/mL were incubated in the presence or absence of 100 ng/mL LPS for 48 h. Cells from each group were then washed, split in different tubes, and incubated with 2 mM 2-deoxy-D-Glucose (2-DG; Cayman Chemical, Ann Arbor, MI) to limit glycolysis, 1 µM Oligomycin A (Cayman Chemical) to inhibit OXPHOS, or 100 µM 6-Aminonicotinamide (6-AN; Cayman Chemical) to inhibit the pentose phosphate pathway (PPP). To evaluate cytokine production, 100 ng/mL LPS challenge were added to the cells 1h after the metabolic modulators. After 24 h incubation, supernatants were collected and stored at -80° C for further measurement of TNF-α and IL-6 using a Cytometric Bead Array (CBA; BD Biosciences) following the manufacturer’s instructions. To measure the phagocytic capacity, endotoxin-tolerant and control PBMCs were incubated for 22 h with the metabolic modulators before a 2.5 h incubation with 0.5mg/mL pHrodo Green *E. coli* BioParticles (Thermo Fisher Scientific). Cells were stained with anti-CD14-BV711 (BD Biosciences) for monocyte identification and pHrodo green median fluorescence intensity was determined by flow cytometry.

#### 2.2.6 Statistical analysis

Normality was evaluated by the Shapiro–Wilk test. Differences between the distinct conditions were evaluated by repeated measures one-way ANOVA followed by Bonferroni posttest or paired Student’s t-test. A p-value < 0.05 was considered significant and all statistical analyses were performed using Graph Pad Prism 9 (GraphPad Software, San Diego, CA).

## 3 Results

### 3.1 Proteomic analysis indicates a metabolic switch in sepsis patients’ PBMCs

Our previous study integrating transcriptomic and proteomic data from different septic patients cohorts at ICU admission described metabolic changes both in mRNA and protein levels (16). To further investigate how sepsis affects cell metabolism, the proteome of sepsis patients’ PBMCs at ICU admission (obtained from (16)) was analyzed together with samples obtained 7 days after enrollment. At D7 there was a lower number of differentially abundant proteins compared to healthy volunteers than previously observed at D0: 241 altered proteins (148 upregulated, 93 downregulated) at D7 (**Supplementary File 2**) compared to 703 (372 upregulated, 331 downregulated) at D0 (16).

**Figure 1:**
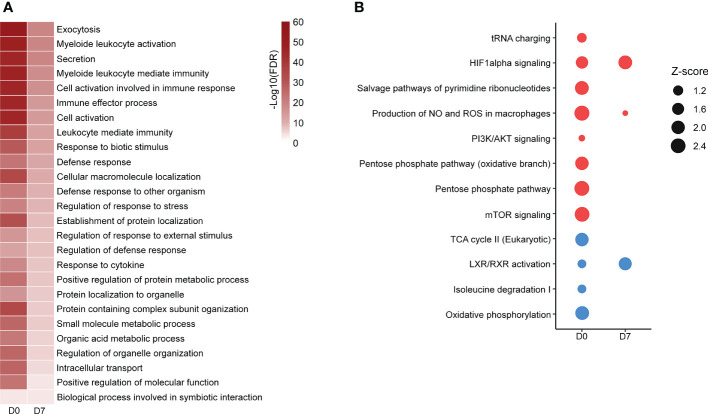
Altered pathways in septic patient’s proteome ad D0 and D7. **(A)**: Gene ontology biological processes significantly enriched at D0 and D7. Color scale represents -Log_10_ (FDR). **(B)**: metabolism-targeted IPA analysis. All selected pathways presented B–H p-value <0.05. Dots size represents the z score, which predicts the activation (red dots) or inhibition (blue dots) of a given pathway. Proteomic data from patients at D0 were obtained from (16).

**Figure 2:**
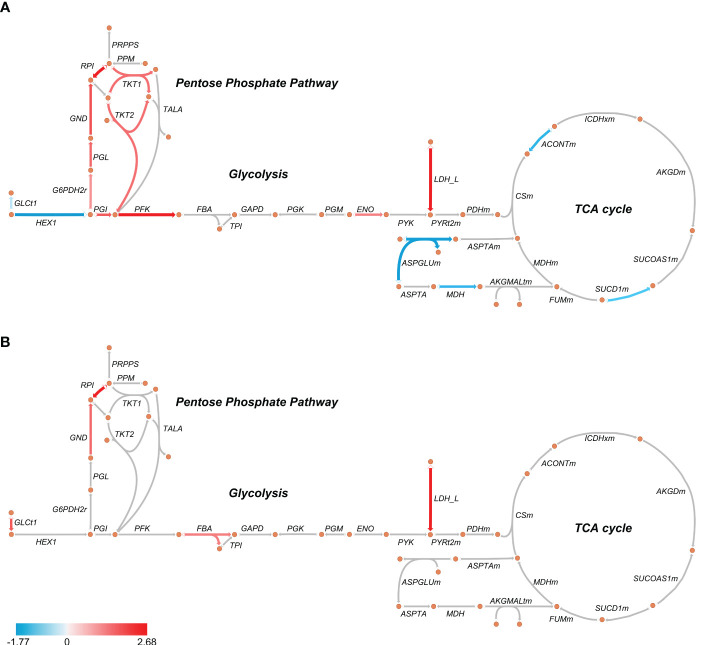
Schematic representation of glycolysis, pentose phosphate pathway, and TCA cycle in septic patients’ PBMCs at D0 **(A)** and D7 **(B)**. The arrows indicate enzymes catalyzing each reaction. Red arrows indicate an average upregulation and blue arrows indicate an average downregulation based on the Log_2_ (fold-change) compared to healthy volunteers. Metabolic maps were generated using Escher.

GOBP analysis showed similar pathways enriched in patients’ PBMCs at D0 and D7 with a reduced number of altered proteins at D7, indicating a return to homeostasis. “Positive regulation of protein metabolic process”, “small molecule metabolic process” and “organic acid metabolic process” were among the enriched processes both at D0 and D7 (**Figure 1A**; **Supplementary File 3**). Metabolism-targeted IPA analysis at D0 indicated activation of PPP, production of ROS and NO, mTOR and HIF-1α signaling along with inhibition of tricarboxylic acid cycle (TCA) cycle, oxidative phosphorylation, and LXR/RXR pathway; at D7 we found alterations only in HIF-1α signaling, production of ROS and NO and in the LXR/RXR pathway, all of them in the same direction (activation/inhibition) as observed at D0 (**Figure 1B**; **Supplementary File 3**). Additionally, upon a specific analysis of glycolysis, PPP, and TCA cycle, we found most differentially abundant proteins in glycolysis and PPP were upregulated in patients at D0, including, for example, lactate dehydrogenase, while the TCA enzymes aconitase and succinate dehydrogenase were downregulated (**Figure 2A**). At D7, only a few proteins remained upregulated in glycolysis and PPP (**Figure 2B**).

### 3.2 Endotoxin-tolerant cells present an impaired lactate production

**Figure 3:**
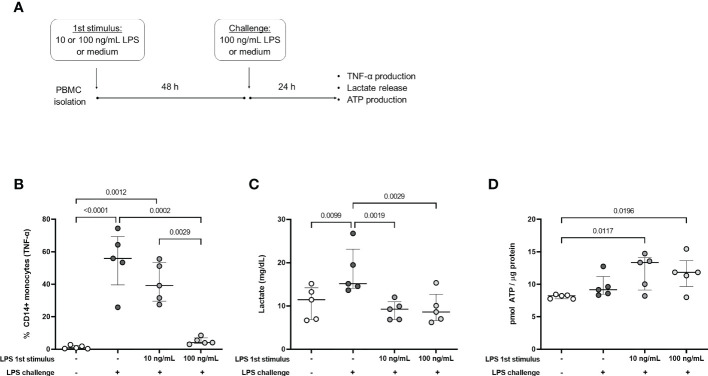
TNF-α production and metabolic profile of endotoxin tolerant PBMCs. **(A)**: PBMCs were incubated for 48h (1st stimulus) with medium (-) or different concentrations of LPS and challenged with 100 ng/mL LPS (+) or medium (-) for 24h. **(B)**: Percentage of CD14^+^ monocytes producing TNF-α, measured by flow cytometry. **(C)**: Lactate concentration in culture supernatants. **(D)**: Intracellular ATP production. Graphs show mean and SD and each dot represents one individual donor evaluated in all experimental conditions (n = 5 donors); p-values indicate differences between each condition as measured by repeated measures one-way ANOVA followed by Bonferroni posttest.

The observed protein content is consistent with an acute response to infection where a metabolic switch to glycolysis and reduction of TCA cycle/OXPHOS are necessary to mount an inflammatory and antimicrobial response (1, 20). Accordingly, in a previous study that enrolled patients from this same cohort, we found increased ROS and NO production in freshly isolated patients’ monocytes. However, upon re-stimulation patients’ cells presented an endotoxin-tolerant profile, with impaired production of inflammatory cytokines and preserved antimicrobial responses (10). Because that study included samples both from community-acquired and hospital-acquired sepsis, we then selected data only from donors included in our proteomic analysis and found the same results (data not shown). To understand how metabolism is linked to this modulation of the immune response we used the endotoxin-tolerance model in healthy-volunteers’ PBMCs (**Figure 3A**), where cells pre-stimulated with LPS (tolerized cells) present an altered immune response similar to sepsis patients (7). In agreement with previous studies (21, 22), cells exposed to 100 ng/mL LPS were unable to produce TNF-α in response to a second stimulus (challenge) (**Figure 3B**). To investigate whether tolerized cells switched their metabolism to glycolysis we measured lactate release in the supernatant under different conditions. Cells exposed only to the challenge dose of LPS produced more lactate than unstimulated cells (**Figure 3C**), which is consistent with an acute response as seen in the sepsis patients’ proteomics at D0. On the other hand, along with the decreased TNF-α production, tolerized cells presented impaired lactate release when challenged with LPS, independent of the pre-stimulus concentration (**Figure 3C**). Additionally, LPS-tolerant PBMCs presented an increased ATP production compared to unstimulated cells (**Figure 3D**).

### 3.3 Glucose metabolism is required to support endotoxin-tolerant leukocytes’ function

**Figure 4:**
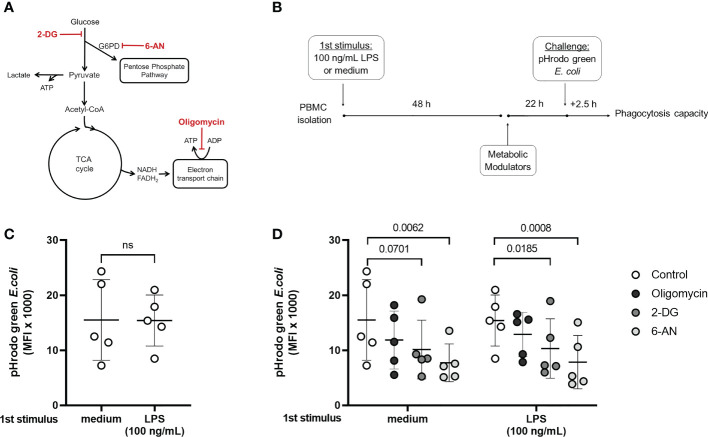
Phagocytic capacity in control and endotoxin tolerant PBMCs is affected by the modulation of cell metabolism. **(A)**: Schematic figure of the inhibition of cell metabolism using 2-DG, 6-AN, and Oligomycin and its specific targets. **(B)**: PBMCs were incubated for 48h (1^st^ stimulus) with medium or 100 ng/mL of LPS, washed, and incubated for 22 h with the metabolic modulators before exposition to pHrodo green *E. coli* particles for 2.5 h. **(C)**: Phagocytic capacity of control (medium) and endotoxin tolerant (100 ng/mL LPS) CD14^+^ monocytes measured as the median fluorescence intensity (MFI) of pHrodo green by flow cytometry. **(D)**: Phagocytic capacity of control and endotoxin tolerant monocytes incubated with metabolic inhibitors. Graphs show mean and SD and each dot represents one individual donor evaluated in all experimental conditions (n = 5 donors); p-values indicate differences between each condition as measured by paired Student’s t-test **(C)** or repeated measures one-way ANOVA followed by Bonferroni posttest **(D)**. ns, not significant.

While endotoxin-tolerant and sepsis patients’ cells present a defective inflammatory cytokine production, other functions such as phagocytosis and ROS productions are maintained (7, 11). In agreement with previous reports (14), the inhibition of lactate production in tolerized cells indicates that they do not favor glycolysis over OXPHOS, which might be related to a suppressed immune response. However, glucose flux can still be necessary to maintain functions that are preserved in endotoxin tolerance; for example, glucose uptake fuel the PPP, important for phagocytosis and pathogen killing (23, 24). Phagocytosis was evaluated in naïve and tolerized cells incubated with 2-DG, 6-AN, and Oligomycin A, to inhibit glycolysis, PPP, and OXPHOS, respectively (**Figures 4A, B**). A pre-incubation with LPS did not affect the phagocytic capacity, confirming that this function is preserved in tolerized cells (**Figure 4C**). The PPP inhibitor 6-AN reduced phagocytosis in both control and endotoxin-tolerant cells, and 2-DG presented the same effect in tolerized cells with a decreased trend in the non-tolerized cells (p = 0.0701) (**Figure 4D**), supporting the idea that glucose metabolism supports endotoxin-tolerant cell function.

**Figure 5:**
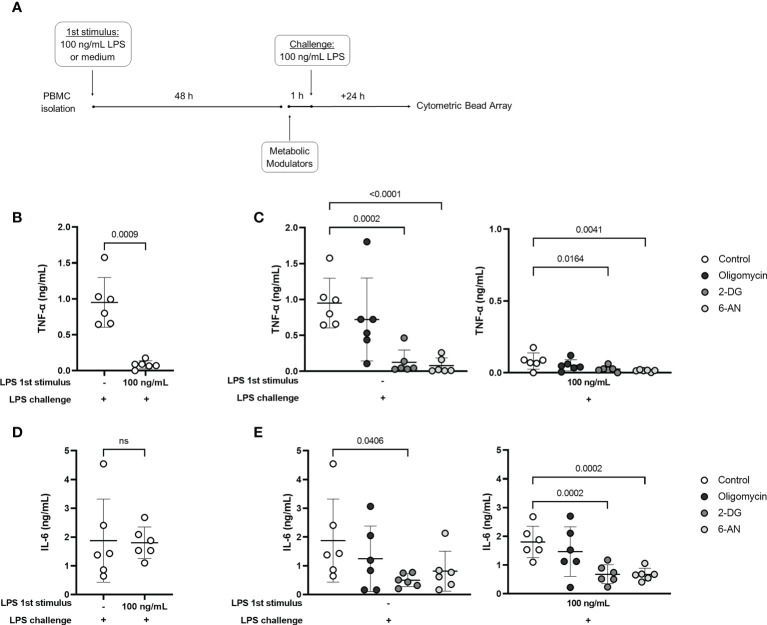
Glucose metabolism supports cytokine production in control and endotoxin tolerant PBMCs. **(A)**: PBMCs were incubated for 48h (1^st^ stimulus) with medium or 100 ng/mL of LPS, washed, and incubated for 1 h with 2-DG, 6-AN, and Oligomycin before challenge with 100 ng/mL LPS. TNF-α **(B)** and IL-6 **(D)** concentration in the culture supernatant of control (-) and endotoxin-tolerant PBMCs challenged with LPS. TNF-α **(C)** and IL-6 production **(E)** by control (-) and endotoxin tolerant PBMCs incubated with metabolic inhibitors along with LPS challenge. Graphs show mean and SD and each dot represents one individual donor evaluated in all experimental conditions (n = 6 donors); p-values indicate differences between each condition as measured by paired Student’s t-test **(B, D)** or repeated measures one-way ANOVA followed by Bonferroni posttest **(C, E).** ns, not significant.

Finally, the release of TNF-α and IL-6 in the culture supernatants was evaluated in control and tolerized cells incubated with the metabolic modulators along with the LPS challenge (**Figure 5A**). As also observed by intracellular measurement (**Figure 3B**), TNF-α production was clearly reduced in tolerized cells (**Figure 5B**), while IL-6 release was preserved (**Figure 5D**). In the control group (non-tolerized cells), incubation with 2-DG and 6-AN reduced TNF-α release, and 2-DG inhibited IL-6 (**Figure 5C, E**) induced by LPS challenge. Interestingly, 2-DG and 6-AN induced a further decrease in LPS-induced TNF-α production in tolerized cells (**Figure 5C**). These metabolic modulators also inhibited IL-6 in the endotoxin-tolerant group (**Figure 5E**). The observed results indicate that despite the inhibition of TNF-α and lactate production, glucose metabolism is important to support functions that are preserved in endotoxin-tolerant cells and to maintain TNF-α response, even if to a lower extent.

## 4 Discussion

Metabolic changes can affect immune responses in sepsis, where immunosuppression and inflammation take place simultaneously. Here, we describe that PBMCs of septic patients present proteomic alterations consistent with a metabolic switch to glycolysis. Upon *ex vivo* stimulation patients’ cells have an endotoxin tolerance phenotype, with impaired cytokine production and preserved antimicrobial responses (10). Using the endotoxin-tolerance model in PBMCs from healthy donors, we found a compromised metabolic adaptation to glycolysis in cells re-stimulated with LPS, which presented inhibited lactate production. Nonetheless, glucose flux was necessary to support phagocytosis and even cytokine production in tolerant cells.

Our proteomic analysis in PBMCs revealed upregulated proteins belonging to glycolysis, PPP, HIF-1α and mTOR signaling, while proteins related to the TCA cycle and OXPHOS were downregulated in patients at ICU admission compared to healthy donors. At D7 we observed fewer enriched pathways, consistent with a return to homeostasis, as most patients in this cohort recovered from sepsis (80%). Contrasting with the proteomic data, a transcriptomic analysis data from patients with bacterial and fungal sepsis also found upregulation of glycolysis, yet together with upregulation of OXPHOS and inhibition of mTOR signaling (14). In addition, we recently reported downregulation of HIF-1α responsive genes in a similar cohort of patients at ICU admission (13). Discrepancies between mRNA and protein expression can be explained by several reasons, including a faster and more transient mRNA response, and posttranscriptional and posttranslational regulations (25, 26). Noteworthy, in our previous work we found HIF inhibitors were downregulated in all patients’ groups, with upregulation of HIF1-α and glycolytic genes in patients who did not survive, especially at D7 samples (13); in the present study, the HIF-1α pathway was the only one with a z-score higher than 2 in the metabolism-targeted IPA analysis (meaning a significantly predicted activation state) at the same time point (D7). The reduced content of proteins implicated in OXPHOS could be a part of the metabolic adaptation but may also be related to mitochondrial dysfunction. Previous studies reported an impaired enzymatic activity of electron transport chain (ETC) complexes I, III, and IV and reduced oxygen consumption in patients’ leukocytes despite a preserved mitochondrial mass (27, 28). Along with that, reduced expression of ETC genes was observed in non-survivor septic patients (29), and we recently described impairment of mitochondrial translation in sepsis (16).

Production of ROS and NO was also identified in the proteomic analysis as an activated pathway in patients’ samples both at D0 and D7, and high levels of ROS were observed in monocytes from patients in the same cohort, together with preserved phagocytosis and indication of increased NADPH oxidase 2 (NOX2) activity (10). Hence, it is possible that the upregulation of PPP proteins in patients’ PBMCs is related to the provision of NADPH supply to sustain ROS production and antimicrobial response (30). Additionally, the increased ROS production in septic patients’ cells could be also a consequence of impaired OXPHOS and mitochondrial dysfunction (31).

PBMCs are mixed cell populations with different metabolic characteristics (32), and in sepsis PBMCs often contain low-density neutrophils (16), which present a highly glycolytic metabolism (33) and could influence our observations. Interestingly, a recent study (34) evaluated metabolic alterations specifically in CD14^+^ monocytes from 9 patients with septic shock and found similar results, with increased glycolytic metabolism and reduced OXPHOS and fatty acid oxidation in samples obtained within 72 h of ICU admission. Therefore, despite the interference of other immune cell populations, our data likely reflect the monocyte phenotype.

It has been described that “immunotolerant monocytes” from septic patients (14) and monocytic cells in *in vitro* models of endotoxin tolerance (15, 35) fail to mount a metabolic response upon stimulation, with amongst other reduced extracellular acidification levels. Accordingly, together with fewer TNF-α-producing monocytes, we found impairment in lactate production in tolerant cells upon LPS challenge. However, limiting glycolysis with 2-DG and the PPP with 6-AN led to impairment of cytokine production and phagocytic capacity both in control and tolerant leukocytes. Glucose metabolism is essential for a proper immune response. It has been shown that 2-DG inhibits LPS induction of TNF-α, IL-6, and IL-1β in PBMCs from healthy volunteers (36), and glycolysis inhibition also impaired phagocytosis in different experimental conditions (37–39). Additionally, blockage of PPP also inhibited TNF-α and IL-6 production in primary macrophages and the RAW 264.7 cell line (40, 41). Overall, our results indicate that glucose metabolism is important not only to mount an immune response in naïve cells but also in supporting endotoxin-tolerant cell function, even the low levels of TNF-α.

Our study has some limitations. The use of PBMCs masks information about specific cell types, however, as previously mentioned, similar results were found with isolated monocytes (34). Also, while proteomic data provide valuable functional information, the addition of other approaches such as fluxomics or phosphoproteomics would help to obtain a more accurate representation of cell metabolism. In the *in vitro* endotoxin-tolerance model, we focused on glucose metabolism, and other metabolic pathways that can affect the immunomodulation were not evaluated, for example, glutaminolysis (42). Finally, the endotoxin tolerance in healthy leukocytes is a simplistic model that does not fully represent the complex events that may influence immune and metabolic response in sepsis, although its similarities are helpful to understand some aspects of sepsis pathophysiology (7).

In summary, our study describes that PBMCs from patients with sepsis presented a phenotype compatible with a metabolic modulation towards glycolysis. In addition, analyses of PBMCs from healthy volunteers in an endotoxin-tolerance model indicate that glucose metabolism supports leukocytes’ functions in a condition of immunomodulation, similar to sepsis, with both preserved (phagocytosis) and suppressed (TNF-α production) cellular responses.

## Data availability statement

Mass spectrometry data from patients at D0 and healthy volunteers were obtained from the ProteomeXchange Consortium via the MassIVE partner repository with the dataset identifier MSV000087733. Additional mass spectrometry data of the 17 D7 patients were deposited to the ProteomeXchange Consortium via the MassIVE repository with the dataset identifier MSV000090356.

## Ethics statement

The studies involving human participants were reviewed and approved by CEP/UNIFESP - Comitê de Ética em Pesquisa da Unifesp - Universidade Federal de São Paulo. The patients/participants provided their written informed consent to participate in this study.

## Author contributions

Conceptualization: RS, BF; investigation: BF, MS, MB; mass spectrometry data acquisition: AT, EN; formal analysis: BF, MS, GL; writing the original draft: BF, RS; review, editing, and revision: BF, MS, GL, MB, AT, EN, TP, RS. All authors contributed to the article and approved the submitted version.

## Funding

This study was supported by FAPESP (2017/21052-0). BF and MS received scholarships from FAPESP (2016/13855-2, 2020/05077-5). GL has a scholarship from FAPESP (2019/20532-3).

## Acknowledgments

The authors thank Edécio Cunha Neto from Instituto do Coração/FMUSP for making available the Ingenuity Pathway Analysis software; and Murillo Assunção, Luciano Cesar Pontes Azevedo, and Flávio Freitas for the support with the selection and enrollment of patients.

## Conflict of interest

The authors declare that the research was conducted in the absence of any commercial or financial relationships that could be construed as a potential conflict of interest.

## Publisher’s note

All claims expressed in this article are solely those of the authors and do not necessarily represent those of their affiliated organizations, or those of the publisher, the editors and the reviewers. Any product that may be evaluated in this article, or claim that may be made by its manufacturer, is not guaranteed or endorsed by the publisher.
