# Supplementary material for: Glucose metabolism is upregulated in the mononuclear cell proteome during sepsis and supports endotoxin-tolerant cell function
Source: Front Immunol. 2022 Nov 18;13:1051514. doi: 10.3389/fimmu.2022.1051514 (PMC9718365; doi:10.3389/fimmu.2022.1051514)
Supplement: Supplementary file 1 [file DataSheet_1.docx]

Supplementary Material

#
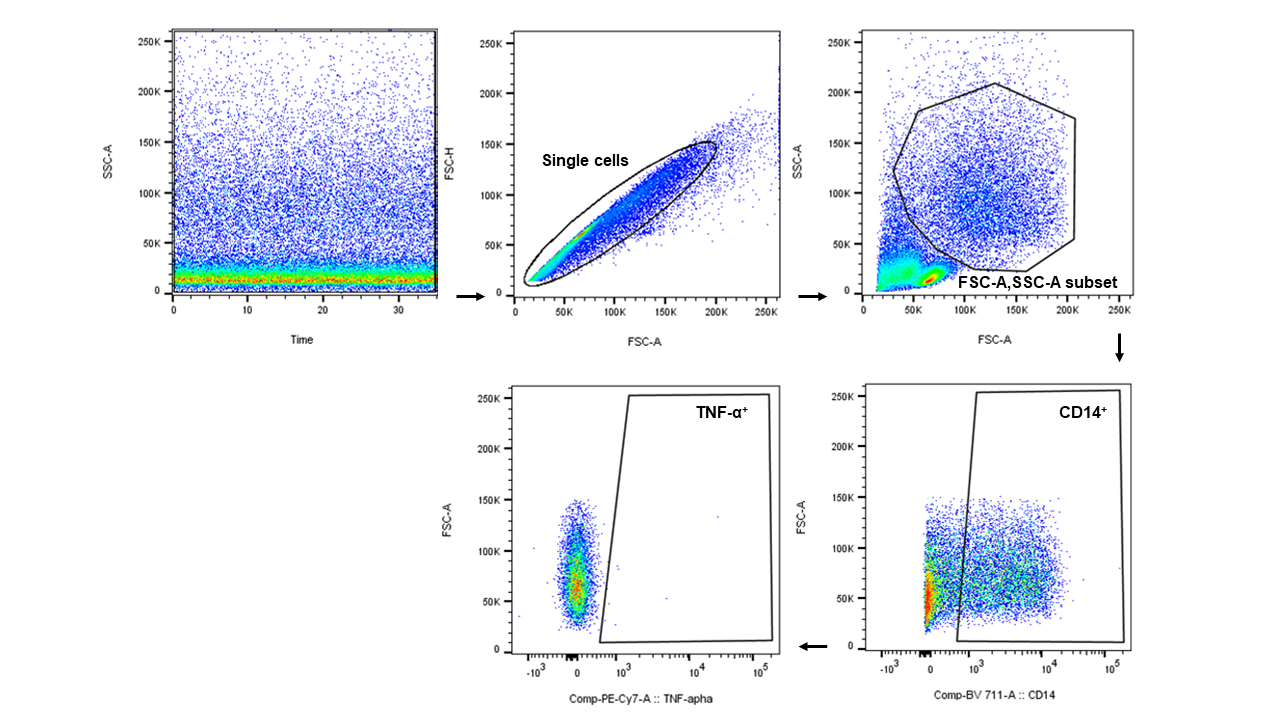
Supplementary Figures

**Supplementary Figure 1.** Gate-selection strategy for flow cytometry analyses. Intracellular TNF-α production was evaluated in CD14^+^ monocytes after a selection of single cells and the removal of debris in the forward versus side scatter gating. The same selection of CD14^+^ monocytes was used to evaluate phagocytosis.
